# Establishment and preliminary evaluation of an RT-ERA combined with CRISPR/Cas12a assay for detection of H5 and H7 influenza viruses

**DOI:** 10.3389/fcimb.2026.1922968

**Published:** 2026-09-16

**Authors:** Zhenghan Luo, Minzhi Xu, Letian Zhang, Yifang Han, Anqi Tan, Taiwu Wang, Fuqiang Ye, Chunhui Wang, Jinhai Zhang

**Affiliations:** 1 Huadong Research Institute for Medicine and Biotechnics, Nanjing, China; 2 Department of Microbiology, Faculty of Naval Medicine, Naval Medical University, Shanghai, China

**Keywords:** CRISPR/Cas12a, ERA-CRISPR/Cas12a, H5, H7, novel influenza A virus, reverse transcription enzymatic recombinase amplification (RT-ERA)

## Abstract

**Background/objectives:**

Novel influenza A viruses (NIAV) infection is a zoonotic acute respiratory disease. The H5N1 and H7N9 subtypes cause high mortality, posing major public health threats. This study aimed to establish rapid, field-deployable subtype-specific nucleic acid detection assays for H5 and H7 by integrating reverse-transcription enzymatic recombinase amplification (RT-ERA) with CRISPR/Cas12a. Two separate reactions are required per sample.

**Methods:**

Conserved hemagglutinin (H) sequences were retrieved from the NCBI database, and primers, probes, and crRNAs were designed using Primer Premier 5 and Primer-BLAST. The sensitivity of RT-ERA alone was compared with that of the ERA-CRISPR/Cas12a using serially diluted virus strain nucleic acid. The specificity was verified against other respiratory pathogens with similar clinical manifestations.

**Results:**

The optimal primers were F1R2 for H5 and F2R3 for H7. The limit of detection (LOD) of RT-ERA alone was 2.63×10³ copies/µL for H5 and 1.86×10³ copies/µL for H7; the ERA-CRISPR/Cas12a assay improved the LOD to 2.63×10¹ copies/µL for H5 and 1.86 copies/µL for H7, representing 100-fold and 1000-fold enhancements in sensitivity, respectively. Specificity testing showed that the assay exclusively detected H5N1 and H7N9 with no cross-reactivity to other tested pathogens.

**Conclusion:**

The ERA-CRISPR/Cas12a method exhibits enhanced sensitivity for H5 and H7 subtypes detection, along with high specificity, rapidity, and minimal equipment requirements. It offers a promising screening tool for NIAV infections.

## 1 Introduction

Novel influenza A virus (NIAV) belong to the family Orthomyxoviridae, genus *Influenza A virus*, serving as a significant zoonotic pathogen that threaten human health. Their genome consists of eight single-stranded negative-sense RNA segments, encoding multiple functional proteins, laying the genetic foundation for rapid viral evolution and mutation (Ito, 2025; Nagoba et al., 2025). Among viral proteins, hemagglutinin (HA) and neuraminidase (NA), the major surface antigenic proteins, govern viral antigenic variation. Based on HA antigenic diversity, influenza A viruses have so far been divided into 19 HA subtypes (Centers for Disease Control and Prevention, 2025). Complex viral lineages generated by the reassortment of different subtypes substantially complicate disease prevention and control. Wild waterfowl and poultry constitute the primary natural hosts of NIAV. Nevertheless, driven by viral evolution, multiple NIAV have crossed species barriers and gained the capacity to infect humans directly (Aldhaeefi et al., 2024; Gao et al., 2025). Globally, as many as 14 NIAV subtypes, including H5, H7, H9, and H10, have been documented to infect humans. Among them, H5 and H7 subtypes are associated with high human mortality and pose a particularly severe public health threat (Munoz et al., 2016; Chou et al., 2025). Given the high mutation rate and frequent intersegmental genetic recombination of the NIAV genome, newly emerging strains constitute a major risk. Such strains may cross the species barriers to infect humans, achieve efficient human-to-human transmission, and ultimately spark pandemics.

In recent years, with the advancement of detection technologies, the emergence of isothermal nucleic acid amplification technology has broken the dependence of traditional polymerase chain reaction (PCR) on high-temperature cycling equipment (Asokan et al., 2025). Enzymatic Recombinase Amplification (ERA) achieves a core isothermal amplification system by modifying, mutating, and screening specific tool enzymes, which are derived from bacteria, viruses, and phages, through techniques such as molecular design, directed evolution, and affinity maturation. This technological innovation enables ERA to rapidly scan and target sequences on template DNA via recombinase-primer complexes under isothermal conditions at 42°C. With the assistance of single-stranded DNA-binding proteins to maintain local single-stranded structures, exponential amplification of trace target nucleic acids can be achieved within 20 minutes (Wu et al., 2024). Featuring no requirement for complex temperature control equipment, rapid reaction kinetics, and high sensitivity, ERA exhibits exhibit enormous application potential in scenarios such as grassroots laboratories and field on-site detection.

Meanwhile, the clustered regularly interspaced short palindromic repeats (CRISPR)/CRISPR-associated protein (Cas) system has sparked a technological revolution in biomedical research and diagnostic fields owing to its programmable nucleic acid recognition and cleavage properties (Xue et al., 2025). Among Cas effectors, nucleases represented by Cas12a specifically recognize target double-stranded DNA (dsDNA) under the precise guidance of guide RNA (crRNA). Upon target sequence matching, Cas12a not only efficiently cleaves the target DNA but also its “collateral cleavage” activity can non-specifically degrade other single-stranded DNA (ssDNA) in the system (Li et al., 2018). Based on this property, reporter DNA labeled with fluorophore-quencher pairs can be designed to convert target-nucleic-acid recognition into a readily detectable fluorescent signal, enabling geometric amplification of the detection readout (Chen et al., 2018). To date, the CRISPR/Cas12a detection system has yielded promising results for nucleic-acid detection of diverse pathogens, exhibiting excellent specificity and sensitivity.

Despite substantial progress of ERA and CRISPR/Cas12a technologies in their respective fields, studies investigating their combined application for on-site detection of NIAV H5 and H7 subtypes remain in the exploratory stage. Given the severe threats posed by H5 and H7 subtypes to human health, as well as the limitations of existing detection assays regarding timeliness and convenience, this study innovatively integrates ERA isothermal amplification with the CRISPR/Cas12a detection system based on the HA conserved sequences of NIAV H5 and H7 subtypes. The aim is to establish a rapid nucleic-acid detection assay with improved sensitivity and specificity, laying a foundation for future development of point-of-care testing platforms.

## 2 Materials and methods

### 2.1 Viruses and plasmids

Viral strains, including influenza A virus (FluA) subtypes H1N1 and H3N2, *Mycoplasma pneumoniae* (MP), adenovirus (Adv), and influenza B virus (FluB), were preserved in the Microbial and Viral Strain Preservation Laboratory of Huadong Research Institute for Medicine and Biotechnics. Nucleic acids from H5N1 and H7N9 viral strains were provided by the Department of Microbiology, Faculty of Naval Medicine, Naval Medical University. The H5 and H7 subtypes positive plasmid standard were synthesized by Suzhou Zixi Biotechnology Co., Ltd.

### 2.2 Main reagents

MiniBEST Viral RNA/DNA Extraction Kit Ver.5.0, 2×One Step PrimeScript™ RT-PCR Kit, B*am*HI and E*co*RI restriction endonucleases were purchased from Takara Co., Ltd.; pUC18 vector was purchased from GenScript Biotech Corporation; Pathogen Nucleic Acid Detection Kit was purchased from Beijing Jinhao Pharmaceutical Co., Ltd.; RT-ERA Amplification Detection Kit was purchased from Suzhou Xianda Gene Technology Co., Ltd.; Cas12a Reaction Kit (fluorescent type) was purchased from Shenzhen Yizhi Biotechnology Co., Ltd.; DL1000 DNA marker was purchased from Yeasen Biotechnology.

### 2.3 Preparation of positive plasmid standards

To select conserved target regions for primer and crRNA design, a total of 50 H5 HA sequences and 50 H7 HA sequences from reported human and avian influenza A virus isolates were downloaded from the NCBI database (https://www.ncbi.nlm.nih.gov). The full list of downloaded HA nucleotide sequences and corresponding strain metadata are provided in **Supplementary Files**. Multiple-sequence alignment was performed using DNAman software to identify conserved regions within the HA genes as target sequences for assay development. Representative conservative reference sequences H5 and H7 were selected for plasmid construction. The pUC18 vector was digested with B*am*HI and E*co*RI restriction endonucleases, and the target sequences were cloned into the linearized vector, followed by sequencing verification. The recombinant plasmids were used as positive plasmid standards, and the plasmid construction map is shown in **Figure 1**.

**Figure 1:**
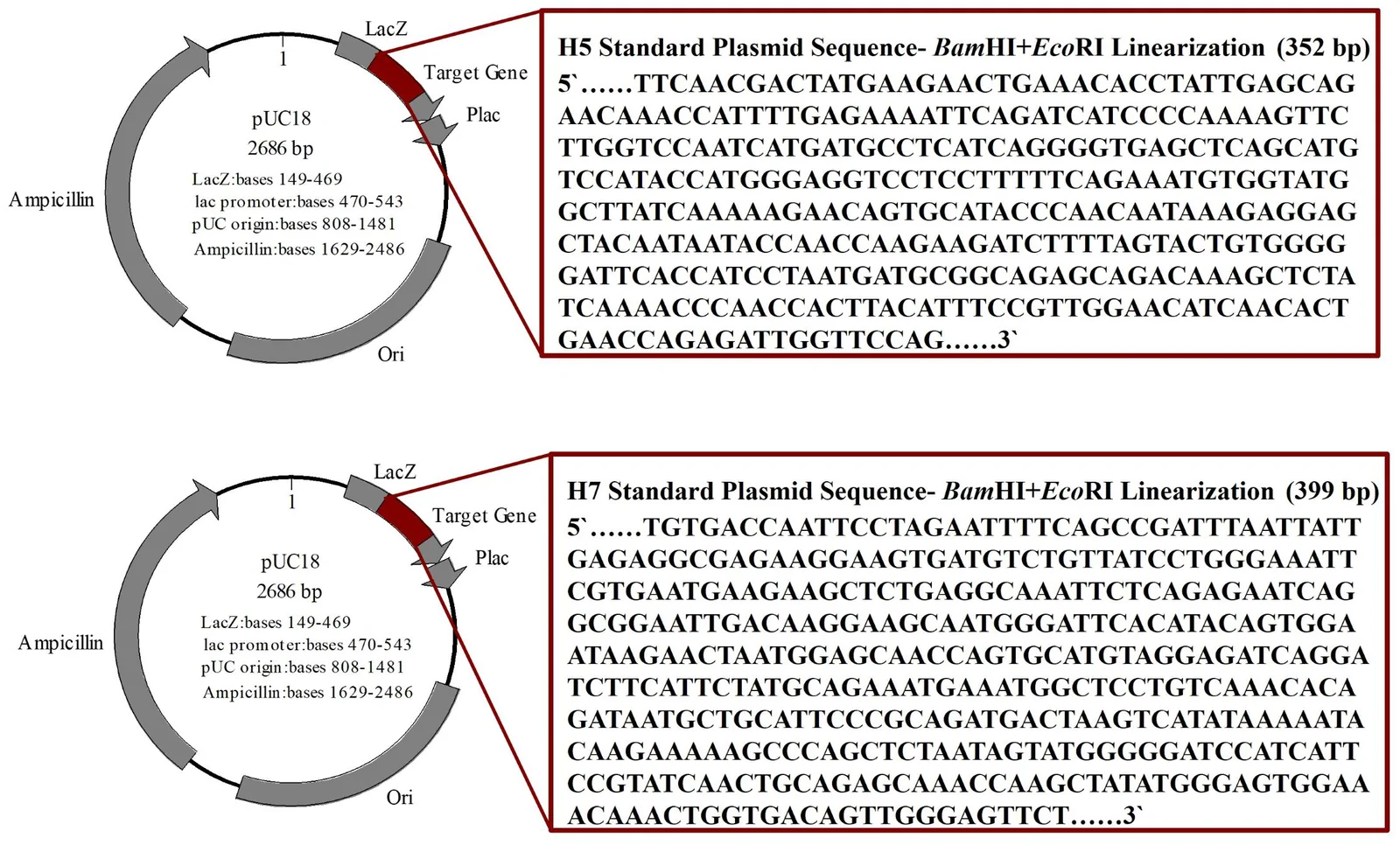
Schematic of H5 and H7 subtypes positive plasmid standard construction.

### 2.4 Design of primers, probes, and crRNA

Primer Premier 5.0 software was used to design and evaluate primer and probe sequences. The design criteria were set as follows: primer length of 17–25 bp, GC content of 40%–60%, amplicon size ranging from 100 to 300 bp, with a tolerance of ±50 bp, and avoidance of stable primer dimers or hairpin structures (evaluated using the OligoAnalyzer tool). For crRNA design, target sequences containing a TTTV PAM site (V = A/G/C) within the conserved regions were selected, and crRNA sequences of 20–24 nt complementary to the target strand were designed. The specificity of all primers was verified using Primer-BLAST against the NCBI nr database. Finally, 2 probe sequences, 9 forward primer sequences, 9 reverse primer sequences, and 2 crRNA sequences were screened out, with the detailed sequences listed in **Table 1**. Some primers with identical sequences were retained in the screening panel to assess primer-design pipeline convergence as well as bidirectional primer activity; initial performance differences were attributable to technical variation. All the aforementioned primers, probes, and crRNAs were synthesized by Suzhou Zixi Biotechnology Co., Ltd.

**Table 1:** Lists of primers, probes and crRNA.

Names	Sequences (5’-3’)	Bases (bp)
H5-crRNA	uaauuucuacuaaguguagauAGAAATGTGGTATGGCTTATCAA	44
H7-crRNA	uaauuucuacuaaguguagauCCTCAGAGCTTCTTCATTCACGA	44
H5-Probe	GTCCTCCTTTTTCAGAAATGTGGTATGGCT(FAM-dT)(THF)(BHQ-dT)CAAAAAGAACAGTGCAT(C3-SPACER)	79
H7-Probe	GGAATAAGAACTAATGGAGCAACCAGTGCA(FAM-dT)(THF)(BHQ-dT)AGGAGATCAGGATCT(C3-SPACER)	77
H5F1	CAACGACTATGAAGAACTG	19
H5R1	**CCAACGGAAATGTAAGT**	17
H5F2	AGATCATCCCCAAAAGT	17
H5R2	**CCAACGGAAATGTAAGT**	17
H5F3	CACCTATTGAGCAGAACAAACCATT	25
H5R3	TTGTAGCTCCTCTTTATTGTTGGGT	25
H5F4	**TGAGCAGAACAAACCATTTTGAGAA**	25
H5R4	**TGAGCAGAACAAACCATTTTGAGAA**	25
H5F5	ACGACTATGAAGAACTGAAACACCT	25
H5R5	TGGTTGGTATTATTGTAGCTCCTCT	25
H7F1	AATGAAGAAGCTCTGAGGCAAATTC	25
H7R1	**TTTGACAGGAGCCATTTCATTTCTG**	25
H7F2	AATTATTGAGAGGCGAGAAGGAAGT	25
H7R2	CAGCATTATCTGTGTTTGACAGGAG	25
H7F3	AGTGATGTCTGTTATCCTGGGAAAT	25
H7R3	**TTTGACAGGAGCCATTTCATTTCTG**	25
H7F4	AATTGACAAGGAAGCAATGGGATTC	25
H7R4	GGAATGATGGATCCCCCATACTATT	25

H5R1/H5R2 and H7R1/H7R3 share identical sequences as they were independently designed from different primer design rounds and were used to evaluate the convergence of the primer design pipeline. H5F4 and H5R4 share identical sequences; this pair was designed to test whether a single sequence could function bidirectionally, but it was not selected as the optimal combination. All sequences were verified by Sanger sequencing of the synthesized oligonucleotides.

Bold values in Table 1 indicate primers with identical sequences. H5R1/H5R2 and H7R1/H7R3 share identical sequences because they were independently designed in different primer design rounds and were retained to evaluate the convergence of the primer design pipeline. H5F4 and H5R4 share identical sequences; this pair was designed to test whether a single oligonucleotide could function as both forward and reverse primers, but it was not selected as the optimal combination. All primer, probe, and crRNA sequences were verified by Sanger sequencing.

### 2.5 Screening of optimal ERA primer pairs for H5 and H7 subtypes

Optimal ERA primer combinations were identified through sequential screening. For H5, reverse primers H5R1-H5R5 were individually tested with a fixed forward primer (H5F1) and probe to select the optimal reverse primer, which was subsequently paired with forward primers H5F1-H5F5 for secondary screening. The final primer set was validated using nuclease-free water as a negative control. An identical strategy was employed for H7. Primer combinations were evaluated based on the following criteria: time to reach the fluorescence threshold (Tt value), endpoint fluorescence intensity, background signal level in the negative control, and the shape of the amplification curve (the presence of a characteristic sigmoidal curve). The combination exhibiting the lowest Tt value, highest endpoint fluorescence, and minimal background signal was selected as the optimal primer set.

### 2.6 Quantification of nucleic acids from H5N1 and H7N9 viral strains

The concentrations of H5N1 and H7N9 positive-control nucleic acids were quantified as 8.84 ng/μL and 6.34 ng/μL, respectively, using a Qubit 4.0 Fluorometer. The initial copy numbers of the H5 and H7 viral nucleic acids were calculated using the following formula: Concentration (copies/μL) = (6.02 × 10²³) × (Con. (ng/μL) × 10^−9^) / (viral nucleic acids length (bp) × 660). The resulting initial concentrations were 2.63 × 10^9^ copies/μL for H5N1 and 1.86 × 10^9^ copies/μL for H7N9 respectively.

### 2.7 ERA-CRISPR/Cas12a amplification reaction

Isothermal amplification was performed using the RT-ERA Amplification Detection Kit, with viral nucleic acids as templates. The 50 μL RT-ERA reaction mixture contained 20 μL solubilizer, 2.1 μL each of forward and reverse primers (10 μM), 0.6 μL fluorescent probe (10 μM; the probe is labeled with a fluorophore and quencher and generates an increase in fluorescence upon specific hybridization to the ERA amplicon, enabling real-time monitoring of the amplification process), 22.2 μL nuclease-free water, 2 μL activator, 1 μL template, and lyophilized reverse transcription reagents. Reactions were incubated at 42 °C for 20 min in a GS8 fluorescent isothermal amplifier with FAM channel fluorescence recorded every 30 s. Subsequently, 1.2 μL Cas12a nuclease (10 μM), 1 μL crRNA (10 μM), and 0.5 μL fluorescent reporter (10 μM; a single-stranded DNA probe labeled with a fluorophore and quencher at its termini, which generates a fluorescence signal upon trans-cleavage by activated Cas12a) were added directly to the RT-ERA reaction mixture, which was then incubated at 37 °C for 10 min in the GS8 instrument. Fluorescence kinetics were continuously monitored throughout both steps. During the RT-ERA stage, the fluorescence signal originates from the probe hybridization to the ERA amplicon; during the Cas12a stage, the fluorescence signal was generated by Cas12a-mediated trans-cleavage of the reporter. The final positive/negative determination was based on the fluorescence increase in the Cas12a stage, which reflects the specific recognition and cleavage activity of Cas12a. Samples were considered positive when the fluorescence increase during the Cas12a stage exceeded 300 units (a threshold established based on the mean fluorescence of negative control reactions plus 10 standard deviations, determined from 20 independent negative control replicates in this study) and showed a characteristic amplification curve; negative results exhibited flat baselines with no significant signal increase.

### 2.8 Agarose gel electrophoresis

Amplification products were analyzed by agarose gel electrophoresis. A 2% (w/v) agarose gel was prepared in 1× TAE buffer (40 mM Tris-acetate, 1 mM EDTA, pH 8.0). For each sample, a 5 µL aliquot of the amplification product was mixed with 1 µL of 6× DNA loading buffer and loaded onto the gel. A 5 µL aliquot of DL1000 DNA marker (Yeasen Biotechnology) was loaded as a molecular weight standard, low molecular weight at bottom. Electrophoresis was performed at 90 V for 40 min in 1× TAE running buffer. The gel was stained with ethidium bromide and visualized under UV light using a gel documentation system (Bio-Rad), with gel exposure conditions set to auto-exposure and an actual exposure time of 6 s.

### 2.9 Sensitivity evaluation of RT-ERA and ERA-CRISPR/Cas12a detection systems

To systematically evaluate the detection sensitivities of the single ERA system and the ERA-CRISPR/Cas12a combined system for H5 and H7 subtypes NIAV, amplification reactions were performed using viral-strain nucleic acids with different dilution gradients. For sensitivity evaluation, viral-strain nucleic acids were serially diluted 10-fold in nuclease-free water to obtain a range of concentrations. For the RT-ERA alone assay, the tested concentration range was 2.63×10²–2.63×10^6^ copies/µL for H5 and 1.86×10²–1.86×10^6^ copies/µL for H7. For the ERA-CRISPR/Cas12a combined assay, additional lower concentration ranges were tested: 2.63×10^0^–2.63×10¹ copies/µL for H5 and 1.86×10^−1^–1.86×10¹ copies/µL for H7. Each concentration was tested in triplicate. In this study, the limit of detection (LOD) was operationally defined as the lowest concentration that yielded a positive amplification result in all three replicates. It should be noted that this operational definition does not represent a statistically validated LOD (e.g., ≥95% detection probability), and the reported LOD values should be interpreted within the context of the current experimental conditions and positivity criteria.

### 2.10 Pathogen nucleic acid extraction and RT-qPCR amplification

Nucleic acids were extracted from five pathogens, including Influenza A virus (H1N1 and H3N2 subtypes), Influenza B virus, *Mycoplasma pneumoniae*, and Adenovirus, with the MiniBEST Viral RNA/DNA Extraction Kit Ver.5.0 according to manufacturer’s instructions. RT-qPCR was used in this study as a reference method to confirm the presence and quality of nucleic acids extracted from the pathogen panel used for specificity testing. RT-qPCR was performed using the 2×One Step PrimeScript™ RT-PCR Kit (Takara) with the following conditions: reverse transcription at 42 °C for 5 min, initial denaturation at 95 °C for 10 s, followed by 40 cycles of 95 °C for 5 s and 60 °C for 34 s. Fluorescence signals were acquired at the 60 °C annealing-extension step.

### 2.11 Specificity of ERA-CRISPR/Cas12a nucleic acid detection

The specificity of RT-ERA and ERA-CRISPR/Cas12a systems was evaluated using nucleic acids from clinically relevant respiratory pathogens: Influenza A (H1N1, H3N2), Influenza B, *Mycoplasma pneumoniae*, and Adenovirus. Nuclease-free water was used as the negative control, and all assays were performed in triplicate.

### 2.12 Statistic analysis

Data are presented as the arithmetic mean ± SD from at least three independent experiments. One-way analysis of variance (ANOVA) was conducted to analysis difference between multiple-group means, with subsequent Bonferroni *post-hoc* testing, via GraphPad Prism 10.1.2. P values < 0.05 were deemed statistically significant.

## 3 Results

In this study, ERA combined with CRISPR/Cas12a technology was applied for nucleic acid detection of H5 and H7 subtypes in separate reaction systems. Standard plasmids were used for screening optimal ERA primers, whereas viral nucleic acids were utilized for subsequent sensitivity and specificity analyses. The schematic workflow is presented in **Figure 2**.

**Figure 2:**
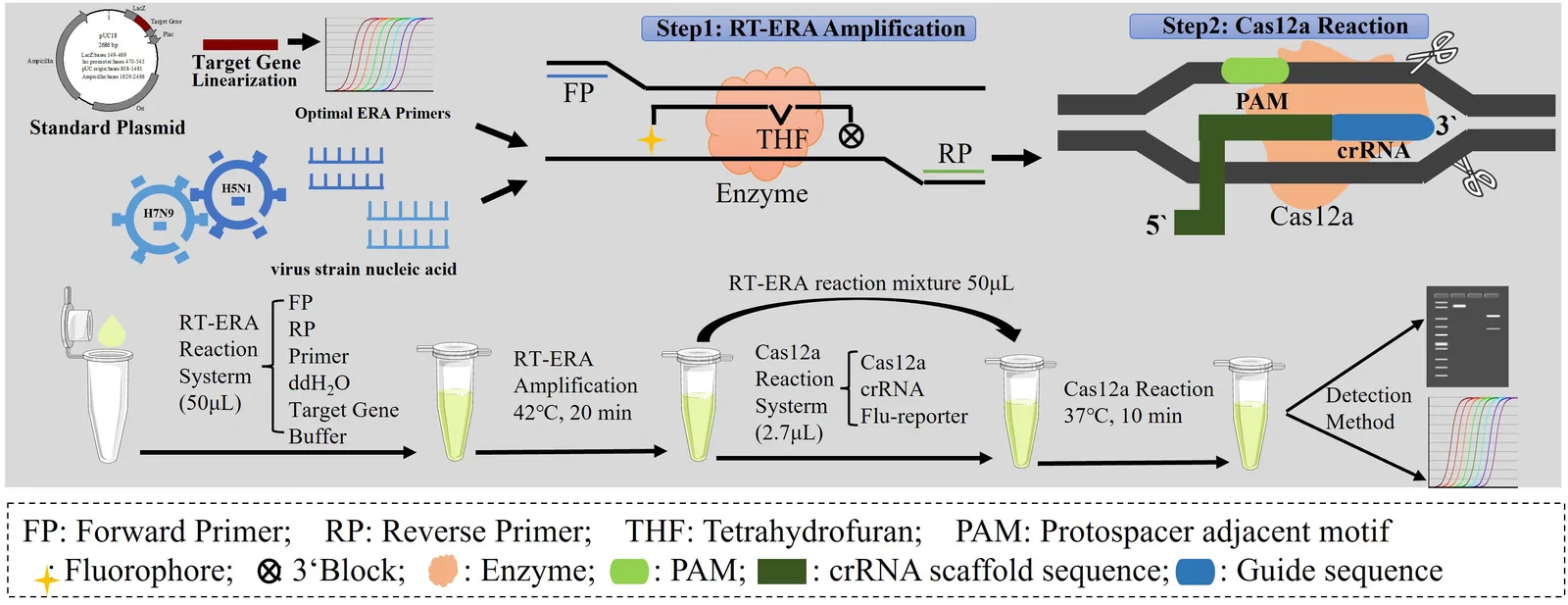
Schematic workflow of the research.

### 3.1 Screening of optimal ERA primers for H5

Negative and positive controls were included in all assays. Forward primer screening identified F1 and F2 as top candidates based on earlier emergence of fluorescence signals (lower Tt values) and higher endpoint fluorescence intensity. Reverse primer screening similarly identified R1, R2, and R3 as the best-performing candidates. During cross-combination testing, the F1R2 pair yielded the earliest amplification signal, the highest endpoint fluorescence and minimal background in the negative control. It was therefore selected as the optimal combination (**Figure 3**).

**Figure 3:**
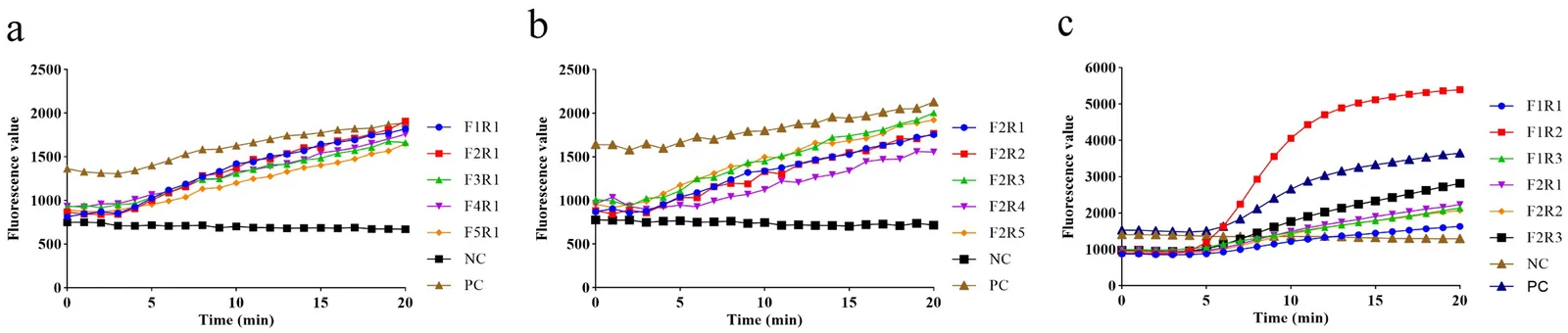
Optimal ERA primers screening for H5. **(a)** Amplification curves of forward primer candidates F1 and F2. **(b)** Amplification curves of reverse primer candidates R1, R2 and R3. **(c)** Amplification curves of optimal primers F1R2.

### 3.2 Screening of optimal ERA primers for H7

Similarly, negative and positive controls were included in the assays. Forward primer screening identified F1 and F2 as the best-performing candidates based on fluorescence signal emergence and intensity. Reverse primer screening similarly identified R1 and R3 as the top candidates. Cross-combination testing revealed that the F2R3 pair produced the earliest and strongest amplification signal with low background. Therefore, this pair was selected as the optimal combination (**Figure 4**).

**Figure 4:**
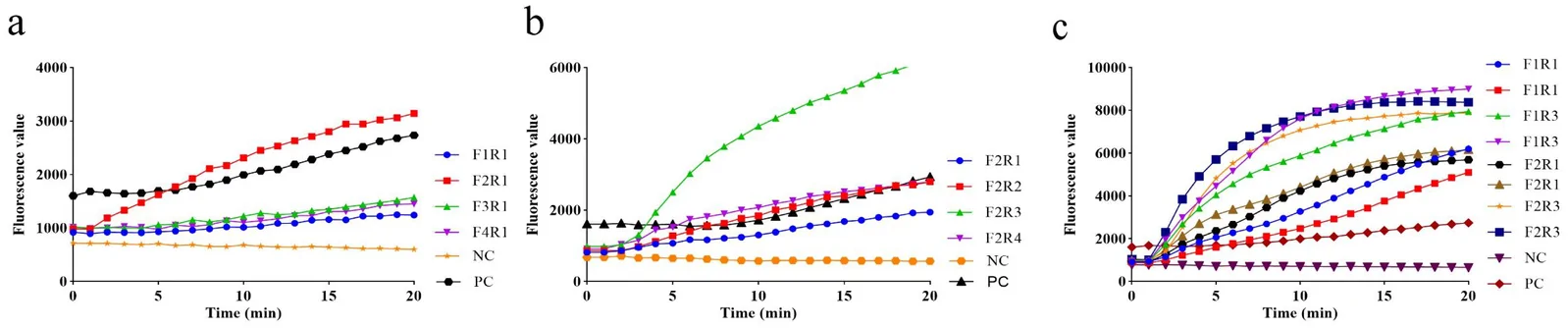
Optimal ERA primers screening for H7. **(a)** Amplification curves of forward primer candidates F1 and F2. **(b)** Amplification curves of reverse primer candidates R1and R3. **(c)** Amplification curves of optimal primers F2R3.

### 3.3 Sensitivity of RT-ERA amplification

Negative, blank, and positive controls were included. Serial 10-fold dilutions of H5N1 viral nucleic acid (2.63×10²–2.63×10^6^ copies/µL) were amplified using the optimal F1R2 primer pair. Positive amplification curves were observed at concentrations of 2.63×10³ copies/µL and above. The lowest concentration yielding a positive result across all three replicates was 2.63×10³ copies/µL, which was therefore defined as the operational LOD of the RT-ERA assay for H5. This result was validated by agarose gel electrophoresis, which yielded a visible band of the expected size (318 bp) at 2.63×10³ copies/µL and above (**Figure 5**).

**Figure 5:**
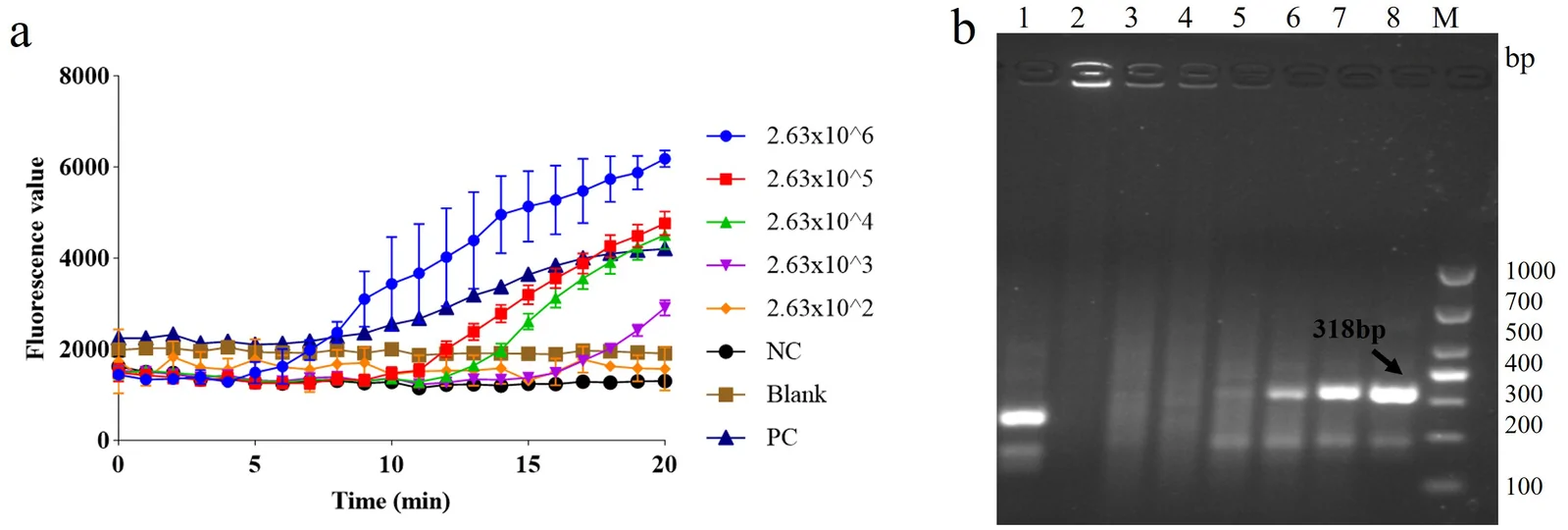
RT-ERA amplification sensitivity assay for H5N1. **(a)** Amplification curves of serial dilutions of the H5N1 viral nucleic acid with single RT-ERA. **(b)** agarose gel electrophoresis of the serial H5N1 amplification product (318 bp). Line 1~8 correspond to PC, Blank, NC, 2.63x10^2^, 2.63x10^3^, 2.63x10^4^, 2.63x10^5^, 2.63x10^6^; M represented Marker; PC, positive control; NC, negative control, including the detection mix, probe&primers and water; Blank, including the detection mix and water, without probe&primers.

Similarly, serial 10-fold dilutions of H7N9 viral nucleic acid (1.86×10²–1.86×10^6^ copies/µL) were amplified with the optimal F2R3 primer pair. Positive amplification was detected at concentrations of 1.86×10³ copies/µL and above, establishing an operational LOD of 1.86×10³ copies/µL for H7. This result was also validated by agarose gel electrophoresis, which yielded a visible band of the expected size (210 bp) at1.86×10³ copies/µL and above (**Figure 6**).

**Figure 6:**
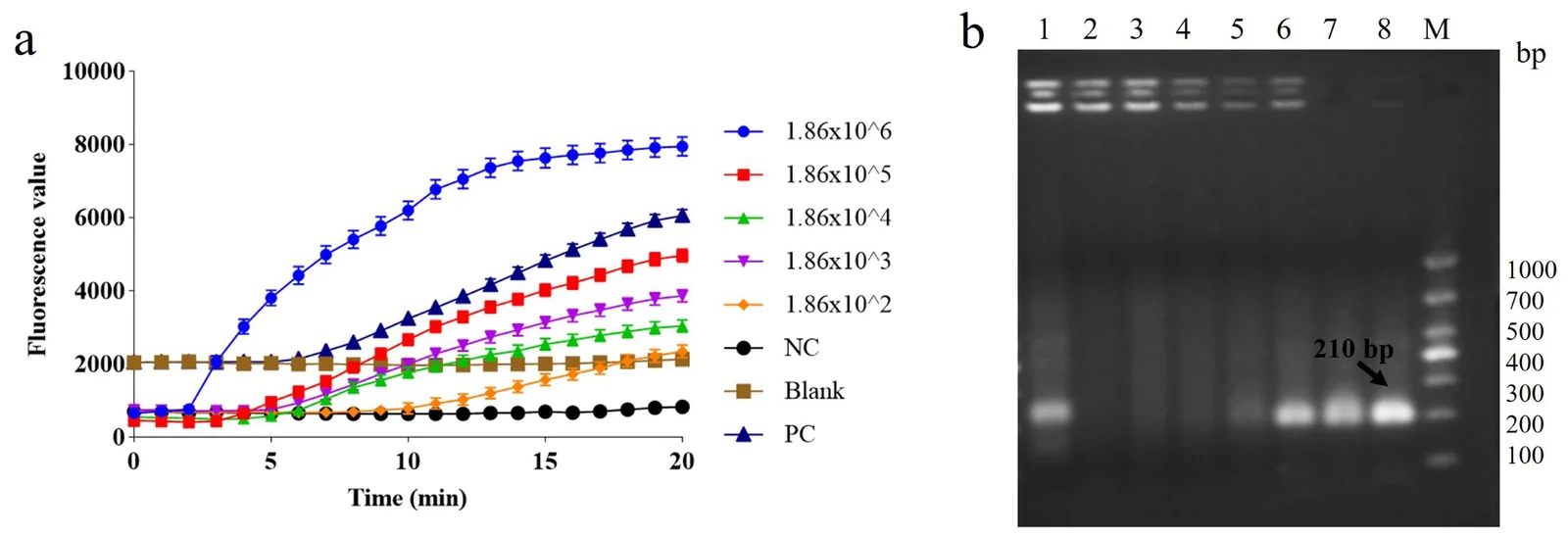
RT-ERA amplification sensitivity assay for H7N9. **(a)** Amplification curves of serial dilutions of the H7N9 viral nucleic acid with single RT-ERA. **(b)** agarose gel electrophoresis of the serial H7N9 amplification product (210 bp). Line 1~8 correspond to PC, Blank, NC, 1.86x10^2^, 1.86x10^3^, 1.86x10^4^, 1.86x10^5^, 1.86x10^6^; M represented Marker; PC, positive control; NC, negative control, including the detection mix, probe&primers and water; Blank, including the detection mix and water, without probe&primers.

### 3.4 Sensitivity of ERA-CRISPR/Cas12a amplification

Sensitivities of standalone RT-ERA assay and the combined ERA-CRISPR/Cas12a system were compared using serially diluted H5N1 and H7N9 viral nucleic acids. For the ERA-CRISPR/Cas12a assay, RT-ERA amplification products generated from serially diluted viral nucleic acids served as substrates for the subsequent CRISPR/Cas12a reaction. For H5N1, positive Cas12a fluorescence signals were observed at nucleic acid concentrations of 2.63×10¹ copies/µL and above. The 2.63×10¹ copies/µL concentration yielded positive results across all three replicates, establishing an operational LOD of 2.63×10¹ copies/µL. This represents a 100-fold improvement relative to the operational LOD of 2.63×10³ copies/µL for RT-ERA alone. For H7N9, positive Cas12a signals were detected at nucleic acid concentrations as low as 1.86 copies/µL, with all three replicates returning positive. At 1.86×10^−1^ copies/µL, inconsistent results were observed, with only one out of three replicates testing positive. Accordingly, the operational LOD was defined as 1.86 copies/µL, representing a 1000-fold improvement relative to the operational LOD of 1.86×10³ copies/µL for RT-ERA alone. It should be noted that the 1.86 copies/µL result is an extremely low copy number and was obtained under controlled experimental conditions using purified viral nucleic acids; hence this value should be interpreted with appropriate caution (**Figure 7**).

**Figure 7:**
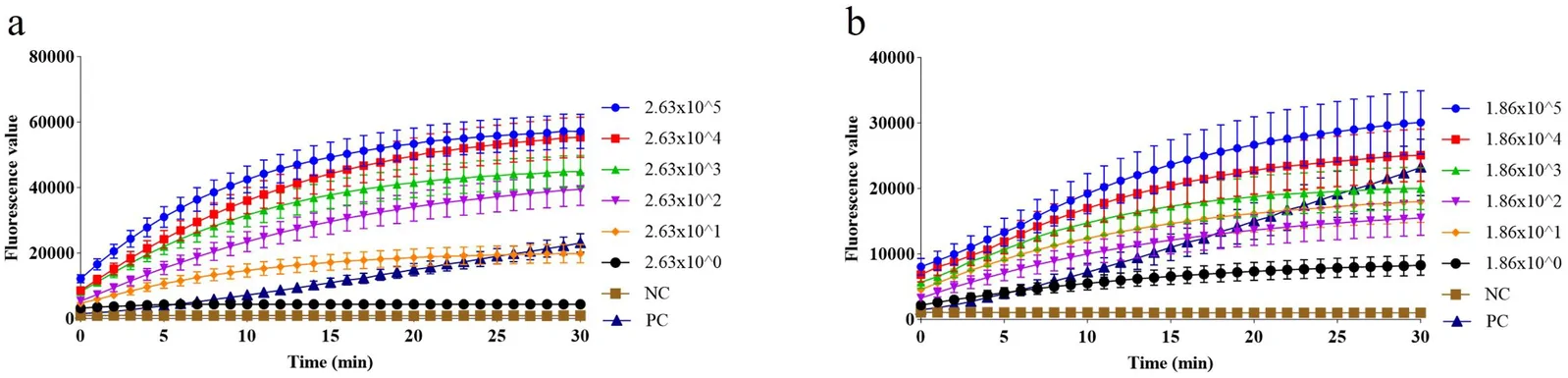
ERA-CRISPR/Cas12a amplification sensitivity assay. **(a)** Amplification curves of serially diluted H5N1 viral nucleic acid with the ERA-CRISPR/Cas12a combined system. **(b)** Amplification curves of serially diluted H7N9 viral nucleic acid with the ERA-CRISPR/Cas12a combined system. H5N1and H7N9 were tested in separate reaction systems.

### 3.5 Specificity of ERA-CRISPR/Cas12a amplification

The specificity of the ERA-CRISPR/Cas12a assay was evaluated against a panel of common respiratory pathogens with similar clinical presentations, including influenza B (FluB), *Mycoplasma pneumoniae* (MP), adenovirus (Adv), and multiple influenza A subtypes (H1N1, H3N2). For the H5 detection system, positive fluorescence signals were observed exclusively for the H5 target, whereas all non-H5 pathogens (including H7N9, FluB, MP, Adv, H1N1, and H3N2) yielded negative results. Similarly, for the H7 detection system, positive signals were observed only for the H7 target, and all non-H7 pathogens tested negative. These results indicate that, for the limited pathogen panel tested, the assay showed no cross-reactivity against the examined non-target respiratory pathogens. Amplification products were further verified by agarose gel electrophoresis (**Figures 8**, **9**).

**Figure 8:**
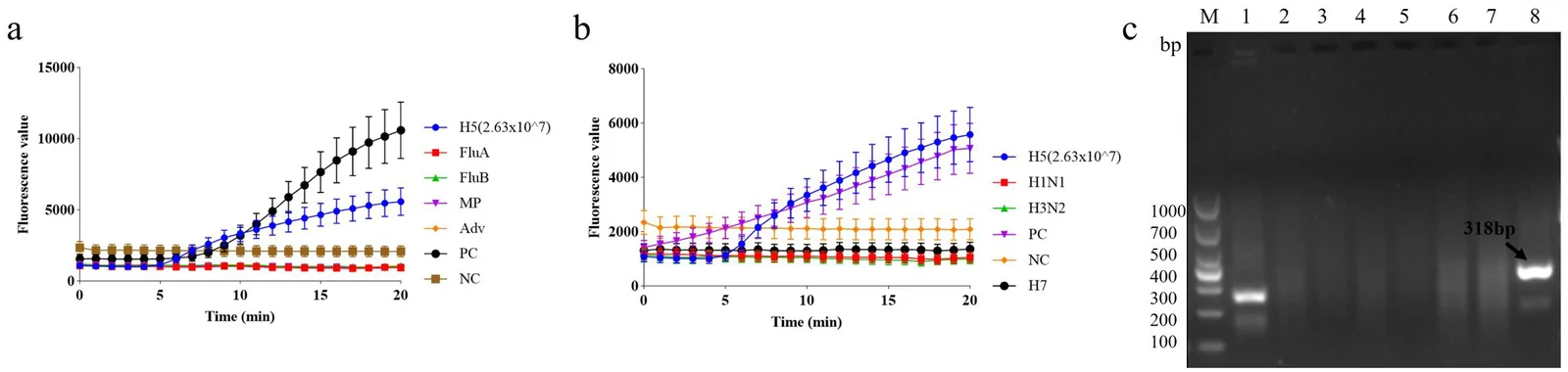
ERA-CRISPR/Cas12a amplification specificity assay for H5N1. **(a)** ERA-CRISPR/Cas12a amplification curves of various respiratory pathogens with H5N1 targets. **(b)** ERA-CRISPR/Cas12a amplification curves of diverse influenza A subtypes with H5N1 targets. **(c)** Agarose gel electrophoresis of amplification products for H5N1 specificity testing. Line 1~8 correspond to PC, H1N1, H3N2, H7N9, FluB, MP, Adv, H5N1; M represented Marker; PC positive control.

**Figure 9:**
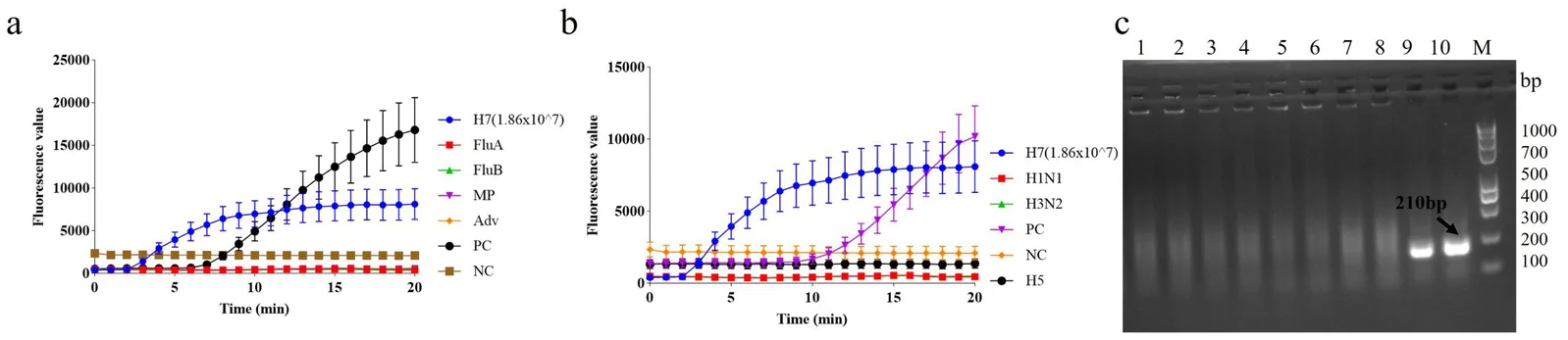
ERA-CRISPR/Cas12a amplification specificity assay for H7N9. **(a)** ERA-CRISPR/Cas12a amplification curves of various respiratory pathogens with H7N9 targets. **(b)** ERA-CRISPR/Cas12a amplification curves of various influenza A subtypes with H7N9 targets. **(c)** Agarose gel electrophoresis of amplification products for H7N9 specificity testing. Line 1~10 correspond to NC, FluA, FluB, MP, Adv, H1N1, H3N2, H5N1, PC, H7N9; M represented Marker; NC negative control; PC positive control.

## 4 Discussion

Although the traditional virus isolation and culture method is the gold standard, it is time-consuming and cumbersome to operate, making it difficult to meet the needs of large-scale rapid screening during outbreaks. Although there are various antigen-antibody detection methods, they have inherent defects such as high false-positive rates, significant differences in the titer of commercial reagents, and long cycles for antiserum preparation. Quantitative PCR technology has significantly improved detection sensitivity and accuracy. However, its dependence on expensive and sophisticated instruments and operational requirements for professional and technical personnel have greatly limited its application and promotion in point-of-care testing (POCT) scenarios (Fu et al., 2023; Asokan et al., 2025). Based on this, this study developed an ERA-CRISPR/Cas12a detection method for the nucleic acids of H5 and H7 subtypes. Through the synergistic effect of isothermal amplification and specific enzyme digestion, it effectively compensates for the shortcomings of traditional detection technologies and provides a new strategy for the rapid detection of NIAV.

CRISPR-Cas12a-powered nucleic acid diagnostics rely on target-triggered collateral single-stranded DNase activity, which provides the molecular basis for signal transduction in our assay (Chen et al., 2018). Benchmarking studies of CRISPR-assisted isothermal detection for influenza and other respiratory viruses have emphasized that performance derived from plasmid templates cannot fully recapitulate assay behavior with authentic viral RNA or clinical specimens, highlighting the necessity of multi-layer validation from synthetic constructs to real viral isolates and clinical samples (Li et al., 2023; Zhou et al., 2024). Many reported CRISPR-influenza detection platforms first optimize primers and crRNAs using plasmid references, then further characterize sensitivity and specificity with viral nucleic acids extracted from authentic viral strains and clinical materials, which enables more realistic assessment of field-oriented diagnostic performance (Shen et al., 2023; Zhou et al., 2024). Consistent with these strategies, standard plasmids were used for screening optimal ERA primers in our study, whereas viral nucleic acids were utilized for subsequent sensitivity and specificity analyses.

In recent years, due to the advantages of no need for temperature cycling equipment and simple operation, isothermal amplification technology has been widely explored in the field of NIAV detection, among which loop-mediated isothermal amplification (LAMP), recombinase polymerase amplification (RPA), and recombinase aided amplification (RAA) technologies are the most extensively applied (Tang et al., 2016; Yang et al., 2023). The real-time quantitative RT-LAMP method established by Golabi et al. can simultaneously detect 9 NIAV subtypes, with the entire process of sample preparation and detection taking only 30 minutes. It can successfully detect spiked viruses in bird swab samples, with a detection limit as low as 10^0.8^ EID50/reaction (approximately 310–483 copies/μL) (Golabi et al., 2021). The paper-based microfluidic LAMP method developed by Kamel et al. has a detection limit of 25 copies/μL for H5 subtype NIAV, further improving the on-site applicability of LAMP technology (Kamel et al., 2025). However, the inherent defect of LAMP technology lies in the need to design multiple pairs of primers, which are difficult to design and prone to non-specific amplification, limiting its detection specificity and application promotion. RAA and RPA technologies have more advantages in primer design difficulty and detection speed. RT-RAA amplification combined with lateral flow dipstick (LFD) technology have a detection limit of 1 copy/μL for H5, with a sensitivity 100 times higher than that of RT-PCR (Yang et al., 2023). However, the single use of isothermal amplification technology generally has the risk of non-specific amplification, which affects the reliability of detection results.

To overcome the limitations of single isothermal amplification technology, the combined strategy of CRISPR/Cas system and isothermal amplification technology has emerged. Through the specific recognition and trans-cleavage activity of Cas proteins on target sequences, the specificity and sensitivity of detection are significantly improved. The H5 detection method established by Zhou Xu et al. using RT-RPA combined with CRISPR/Cas12a technology allows direct observation of results via blue light, with a detection limit of 1.9 copies/μL and good specificity (Zhou et al., 2024). The RAA combined with CRISPR-Cas13a and LFD detection system developed by Yang Li et al. can complete detection within 40 minutes at 37°C, with a sensitivity as high as 0.1 copies/μL (Li et al., 2023).

In the present study, the integrated ERA-CRISPR/Cas12a detection system completed the amplification and Cas12a detection steps within 30 min (20 min for ERA amplification and 10 min for Cas12a trans-cleavage). It should be noted that this 30-min timeframe does not include the time required for sample collection, nucleic acid extraction, and reaction setup. The operational LOD of the combined system was 2.63×10¹ copies/µL for H5 and 1.86 copies/µL for H7 using viral nucleic acids. The detection limit for H5 (2.63×10¹ copies/µL) is on the same order of magnitude as the RT-RPA-Cas12a method reported by Zhou et al. (1.9 copies/µL) (Zhou et al., 2024), though direct quantitative comparison is complicated by differences in target sequences, template types, and detection formats. The H7 LOD of 1.86 copies/µL represents a very low concentration level, and its stability across different experimental conditions requires further investigation. Compared with the RAA-Cas13a-LFD method reported by Li et al. (0.1 copies/μL) (Li et al., 2023), the present system benefits from Cas12a’s more flexible PAM sequence requirements (TTTV) compared with Cas13a’s restrictive PFS requirements, potentially offering broader target sequence versatility. Moreover, we demonstrated that the ERA-CRISPR/Cas12a system enhanced H5 and H7 detection sensitivity by 100-fold and 1000-fold, respectively, compared with standalone RT-ERA. This improvement likely stems from the excess target nucleic acids generated by isothermal amplification, which provide sufficient binding sites for full activation of Cas12a trans-cleavage activity, enabling enhanced reporter probe cleavage and signal generation. This synergistic cascade combines exponential amplification with specific Cas12a cleavage, thereby surpassing the sensitivity limits of single-platform technologies.

Several important limitations of this study should be acknowledged. First, the specificity evaluation was limited to a panel of five non-target respiratory pathogens (H1N1, H3N2, influenza B, Mycoplasma pneumoniae, and adenovirus). While no cross-reactivity was observed within this panel, this does not constitute comprehensive validation of “high specificity.” Expanded testing against a broader range of respiratory pathogens, as well as different clades/subclades of H5 and H7 viruses, is warranted. Second, the current assay workflow requires opening the reaction tube after RT-ERA amplification to add Cas12a reagents, which poses a potential risk of aerosol contamination given the high concentration of amplification products. Multiplexed one-pot CRISPR diagnostic workflows represent an important developmental direction for point-of-care nucleic acid testing, yet they face well-documented technical bottlenecks including primer-primer interference, amplification competition among multiple targets, and reporter crosstalk, which may compromise low-copy detection performance and increase contamination risks if not properly optimized (Ding et al., 2020; Xie et al., 2025). Multiple strategies have been reported to achieve simultaneous multi-target detection, including spatial compartmentalization on microfluidic cartridges and orthogonal Cas-reporter systems in homogeneous one-pot reactions (Ding et al., 2020; Fan et al., 2024). Future work will explore whether our current separate-tube H5- and H7-detection reactions can be transformed into a closed-tube multiplex format, while preserving the high analytical sensitivity observed in this study. Third, the present study relies on a benchtop fluorescence isothermal amplifier for signal readout; portable readers or visual detection formats have not yet been implemented. Therefore, the current system should be regarded as a laboratory-based method with potential for future POCT adaptation rather than a mature field-deployable platform. Microfluidic biosensing platforms provide feasible solutions for integrating sample lysis, nucleic acid release, amplification and CRISPR detection into compact sample-to-answer devices, which greatly simplifies manual operation and reduces aerosol contamination risks for field-deployment diagnostics (Li et al., 2026).

The core features of the current system are: (1) isothermal operation (37–42 °C) without the need for a thermal cycler; (2) substantially improved detection sensitivity compared with standalone RT-ERA; (3) fluorescence-based readout using a portable isothermal amplifier; and (4) no cross-reactivity observed with the limited respiratory pathogen panel tested, indicating good preliminary specificity.

In conclusion, this study preliminarily established an RT-ERA-CRISPR/Cas12a detection method for NIAV H5 and H7 subtypes, which showed substantially improved sensitivity compared with RT-ERA alone and demonstrated good specificity in a limited pathogen panel using viral nucleic acids. However, its practical application value requires further evaluation through validation with clinical specimens, expanded specificity testing against diverse viral clades, and field testing. Future research should focus on: (1) Construction of a pseudovirus standard panel and validation with clinical specimens; (2) Development of an integrated microfluidic chip for multiplexed target detection in a closed format to minimize contamination risk; (3) Integration with an intelligent fluorescence reader or lateral flow dipstick for instrument-free visual readout to enhance quantitative capability; (4) Establishment of a rapid crRNA design platform to counter viral mutation challenges and ensure coverage of circulating strains. Through deep integration of technological innovation with prevention and control strategies, this approach may provide useful technical support for advancing frontline prevention of emerging infectious diseases and for strengthening the “One Health” system.

## Conclusion

This study preliminarily established an RT-ERA-CRISPR/Cas12a detection assay for NIAV H5 and H7 subtypes, with each subtype detected in a separate single-channel reaction. With viral nucleic acids extracted from authentic H5N1 and H7N9, this combined assay exhibited substantially improved analytical sensitivity relative to standalone RT-ERA, with a 100-fold improvement for H5 and a 1000-fold improvement for H7. No cross-reactivity was observed against the limited panel of respiratory pathogens tested. The amplification and Cas12a-mediated detection steps can be completed within 30 min under isothermal conditions. Further validation using clinical specimens, expanded specificity testing, and development of integrated closed-tube detection formats are required to evaluate the practical applicability of this method for point-of-care settings.

## Data Availability

The original contributions presented in the study are included in the article/Supplementary Material. The H5 and H7 HA sequence alignment data have been deposited in Zenodo and are publicly available at: https://doi.org/10.5281/zenodo.22283342. All primer, probe, and crRNA sequences designed in this study are listed in **Table 1**. The publicly available HA sequences used for conserved region analysis were obtained from the NCBI GenBank database, and their accession numbers are provided in the **Supplementary Materials**.
