# Supplementary material for: Establishment and preliminary evaluation of an RT-ERA combined with CRISPR/Cas12a assay for detection of H5 and H7 influenza viruses
Source: Front Cell Infect Microbiol. 2026 Sep 16;16:1922968. doi: 10.3389/fcimb.2026.1922968 (PMC13623710; doi:10.3389/fcimb.2026.1922968)
Supplement: Supplementary file 1 [file Table1.docx]

1. H5 standard plasmid sequence: 352bp, pUC18 vector

TTCAACGACTATGAAGAACTGAAACACCTATTGAGCAGAACAAACCATTTTGAGAAAATTCAGATCATCCCCAAAAGTTCTTGGTCCAATCATGATGCCTCATCAGGGGTGAGCTCAGCATGTCCATACCATGGGAGGTCCTCCTTTTTCAGAAATGTGGTATGGCTTATCAAAAAGAACAGTGCATACCCAACAATAAAGAGGAGCTACAATAATACCAACCAAGAAGATCTTTTAGTACTGTGGGGGATTCACCATCCTAATGATGCGGCAGAGCAGACAAAGCTCTATCAAAACCCAACCACTTACATTTCCGTTGGAACATCAACACTGAACCAGAGATTGGTTCCAG

H5F1: CAACGACTATGAAGAACTG

H5R2: CCAACGGAAATGTAAGT

Amplification size: 318 bp

1. H7 standard plasmid sequence: 399bp, pUC18 vector

TGTGACCAATTCCTAGAATTTTCAGCCGATTTAATTATTGAGAGGCGAGAAGGAAGTGATGTCTGTTATCCTGGGAAATTCGTGAATGAAGAAGCTCTGAGGCAAATTCTCAGAGAATCAGGCGGAATTGACAAGGAAGCAATGGGATTCACATACAGTGGAATAAGAACTAATGGAGCAACCAGTGCATGTAGGAGATCAGGATCTTCATTCTATGCAGAAATGAAATGGCTCCTGTCAAACACAGATAATGCTGCATTCCCGCAGATGACTAAGTCATATAAAAATACAAGAAAAAGCCCAGCTCTAATAGTATGGGGGATCCATCATTCCGTATCAACTGCAGAGCAAACCAAGCTATATGGGAGTGGAAACAAACTGGTGACAGTTGGGAGTTCT

H7F2：AATTATTGAGAGGCGAGAAGGAAGT

H7R3：TTTGACAGGAGCCATTTCATTTCTG

Amplification size: 210 bp
